# Beyond the skin: Lived experiences and coping strategies of psoriasis patients in Malaysia

**DOI:** 10.1371/journal.pone.0329066

**Published:** 2026-01-16

**Authors:** Che Jusoh Che Awanis, Mohd Noor Norhayati, Zainab Mat Yudin

**Affiliations:** 1 Department of Family Medicine, School of Medical Sciences, Universiti Sains Malaysia, Kubang Kerian, Kelantan, Malaysia; 2 School of Dental Sciences, Universiti Sains Malaysia, Kubang Kerian, Kelantan, Malaysia; Istituto Dermopatico dell'Immacolata (IDI)-IRCCS, ITALY

## Abstract

**Background:**

Psoriasis is a chronic autoimmune skin condition that significantly impacts an individual’s quality of life, resulting in physical discomfort, psychological distress, and compromised social well-being. However, there is limited understanding regarding the challenges faced by patients in Malaysia. This study examines the lived experiences of patients with psoriasis in Malaysia, focusing on the emotional, social, financial, and treatment-related challenges they face, as well as the coping mechanisms they employ.

**Methods:**

A qualitative, phenomenological approach was employed among members of the Psoriasis Association Malaysia. Purposive sampling was used to recruit adult participants who were capable of participating in the online interview. Data collection involved the use of Google Forms, which included the Malay version of the Dermatology Life Quality Index questionnaire, supplementing the qualitative findings. This was followed by semi-structured online interviews conducted via video conferencing. Thematic analysis was conducted using NVivo version 14, and descriptive analysis was performed using SPSS version 28.

**Result:**

This study involved 30 respondents with a mean age of 44 years diagnosed with psoriasis. The mean (SD) for the duration of illness is 21.3 (11.8) years. About 70% respondents reported that psoriasis had a moderate to very high impact on their quality of life. Thematic analysis has identified six major themes, including physical devastation, emotional burden, disruption in social functioning, treatment hurdles and advancements, financial barriers, and behavioral adaptation.

**Conclusion:**

Psoriasis imposes complex challenges that extend beyond physical symptoms, affecting emotional well-being, social interactions, financial stability, and treatment struggles. In response to the various challenges that arose, respondents developed behavioral adaptations to achieve a better quality of life. Framed within the biopsychosocial model, the findings emphasize the need for a holistic, patient-centred approach to psoriasis care that integrates medical treatment with psychological support and social interventions to improve overall quality of life.

## Introduction

Psoriasis is a chronic autoimmune skin condition characterized by the rapid growth of skin cells, leading to thick, silvery scales and itchy, dry, and red patches on the skin. This condition can be physically uncomfortable and emotionally distressing for those affected, impacting individuals’ quality of life (QoL) [1]. The worldwide burden of psoriasis is substantial, with an estimated 125 million people affected, accounting for approximately 2–3% of the total population [2]. The Malaysia Psoriasis Registry reported 23,803 psoriasis patients from 34 dermatological centers between October 2007 and December 2019 [3]. The annual prevalence and incidence of psoriasis in 2010 and 2020 increased steadily from 0.27% to 0.51% and from 27.8 to 60.9 per 100,000 person-years, with Chinese and Malays consistently having the highest incidence and prevalence, while males had a higher incidence than females [4].

Psoriasis is more than a skin condition. It is associated with significant impairments in physical health, mental well-being, and social interactions. Approximately 38% of adult patients experience a substantial impact of psoriasis, including physical effects that manifest as the development of psoriatic arthritis (PsA), which affects 30% of patients, with inflamed, scaly patches on the skin [5]. The emotional impact of psoriasis extends far beyond its physical symptoms, profoundly affecting individuals’ self-esteem, mental health, and overall QoL. For example, it can contribute to feelings of embarrassment, self-consciousness, depression, isolation, and reduced self-esteem due to visible skin changes [6]. Moreover, the influence of psoriasis on social aspects profoundly impacts the QoL of a patient. Patients with psoriasis may experience societal stigmatization and discrimination due to misconceptions [7].

The economic impact involves patients with psoriasis, given ongoing medical care, treatments, consultation, and potential hospitalization [8]. In addition, managing psoriasis poses treatment challenges, particularly for patients with moderate to severe psoriasis, as traditional treatments like topical medicines and phototherapy may not consistently yield positive results [9].

Patients with psoriasis commonly employ coping strategies such as active coping, acceptance, and positive reinterpretation to navigate the complexities of their condition, leading to improved health outcomes and enhanced QoL over time [10]. Additionally, patients may utilize emotion-focused coping mechanisms like seeking social support and information or engaging in self-blame or avoidance to manage emotional distress and challenges posed by psoriasis [11].

The biopsychosocial model was employed in this study to gain a deeper understanding of the impact of life on patients with psoriasis, which encompasses three key components: biological, psychological, and social. Limited qualitative information about the experiences of psoriasis patients, their effects on life, or patient coping mechanisms has been published in Malaysia. In several quantitative studies, most patients reported a significant impact on QoL [7,12,13]. This study aimed to explore the lived experience of patients with psoriasis in Malaysia, specifically health-related QoL, including emotional, social, financial, and treatment-related challenges faced by psoriasis patients, as well as the coping mechanisms employed by these patients. The QoL is a comprehensive concept encompassing all factors that affect an individual’s life [14].

## Methods

### Setting and participants

A qualitative, phenomenological approach was conducted among patients with psoriasis who were registered with the Psoriasis Association Malaysia. Respondents over 18 years old, diagnosed with psoriasis for more than six months, regardless of severity, and capable of doing online interviews were included. Those with a history of being diagnosed with psychiatric disorders or unable to communicate in the Malay Language or English were excluded. Purposive sampling was applied until thematic saturation was reached.

### Research tool

A semi-structured interview guide was used to cover QoL involving the psychological, emotional, social, financial, and treatment challenges, as well as coping mechanisms, using open-ended questions. It is utilized to elaborate on their lived experience and explore the probing question in depth. Along with the interview, the Malay version of the Dermatology Life Quality Index (DLQI), which is a disease-specific measure regarding QoL, was used with the author’s permission (license ID: CUQoL2419) [15]. It is a validated questionnaire used to triangulate the qualitative study with a Cronbach’s Alpha of 0.844. It consists of a ten-item questionnaire that assesses the impact of dermatological conditions on various aspects and domains, such as symptoms and feelings, daily activities, leisure, work and school, personal relationships, and treatment [12]. The sum of the scores produces a total score between 0 and 30, with higher scores indicating greater impairment in QoL [16]. The outcome from the DLQI complements and strengthens the accuracy of the findings from the in-depth interview.

### Data collection

An in-depth interview was conducted in November and December 2024. The Psoriasis Association Malaysia contacted the eligible patients, and those who agreed to share their experiences provided verbal consent for the interview. Before the interview, the patients were contacted via phone for an introduction to the study. They completed the Google form, which consisted of consent for the research and the Malay version of the DLQI, in five to ten minutes, and it was sent via WhatsApp. An in-depth interview was conducted virtually via video call-facilitated platforms, such as Zoom or Webex, lasting between 45 minutes and one hour.

The conversation was recorded and transcribed verbatim in the respondent’s language. Any missing information was reviewed and updated using the probing and follow-up questions to elicit further detail, while unclear information was clarified with the respondent.

### Data analysis

The verbatim transcripts were managed using computer-aided qualitative data analysis (CAQDA) software, NVIVO version 14 (QSR International Pty Ltd., 2012), using thematic analysis [17]. Data analysis was conducted concurrently with data collection and continued until saturation was achieved. The respondents were identified by identification numbers to maintain the confidentiality of the findings. Descriptive statistics were used to analyze the QoL items using SPSS version 28.

Thematic data analysis, a process of encoding information, was conducted in six phases [18]: (i) Familiarization with the data: involved reading and re-reading the transcripts to ensure researchers understood the overall meaning of the information. (ii) Generating initial codes: the entire dataset was coded, and labels were generated for important information. The initial coding framework was developed by synthesizing concepts identified in previous studies. As the process unfolded, new codes that emerged were expected and compared with the initial ones. (iii) Searching for themes: the codes were merged to form subthemes (subcategories), which were then summarized to create themes (categories). (iv) Reviewing themes: at times, themes were divided, merged, or eliminated throughout the process. (v) Defining and naming themes: the categories or themes were interpreted to derive their meanings. (vi) Producing the report involved integrating the analytical narrative with selected vivid examples to illustrate key points.

Rigor is attained by implementing reliability and validity in the qualitative inquiry [19]. Reliability was ensured in this study by auditing the transcripts and cross-referencing them with the audio recordings to verify that no errors were introduced during transcription and coding. During coding, data were constantly compared with codes to ensure no changes occurred to the meaning of the codes. The coding for the same text was thoroughly reviewed and approved by a co-investigator. Transcripts were also examined for data segments that did not fit the pre-specified codes or the emerging thematic structure. These discrepant cases were reviewed in a team discussion and emerged as the new themes and subthemes. All data were finalized in a thematic structure presented with themes and subthemes to ensure that the analytic framework remained closely aligned with the full range of participants’ experiences.

Validation is achieved in this study through the triangulation of multiple sources, thereby strengthening the credibility of the research. The DLQI questionnaire supported the primary data from the interview by cross-referencing the findings, thereby enhancing the credibility of the information. Next, the member checking process was conducted through phone calls to ensure accuracy, and the refined transcript content and cited quotations were read to the respondents. Rich and thick description was used to present multiple perspectives on a theme, ensuring that the findings were more realistic and enriched. Negative or discrepant information was used to add credibility to the account.

### Ethical approval

This study was conducted in accordance with the principles outlined in the Declaration of Helsinki. Ethical approvals were obtained from the Human Research Ethics Committee, Universiti Sains Malaysia (USM/JEPeM/KK/24040352) (Supplementary 1). Measures were taken to protect the respondent’s vulnerability. Participation was voluntary, with the option to withdraw at any time. Risks, confidentiality, and withdrawal procedures were outlined in the consent form. Surveys were kept anonymous, and interview recordings excluded any identifiable information, which was used only for transcription purposes. Respondents were assigned identification numbers to ensure confidentiality, and personal details remained anonymous. Submitted forms contained no identifying information, and non-participants’ details were not disclosed. Data were entered into SPSS 28 and NVIVO 14, accessible only to the research team and presented as group data.

## Results

### Sociodemographic and clinical characteristics

Thirty respondents were interviewed. Table 1 describes the characteristics of the respondents, 70% of whom were diagnosed with plaque psoriasis, plaque psoriasis with PsA, and plaque with guttate psoriasis, with a mean age of 44 years. The majority of respondents were of Malay ethnicity (43.3%), followed by Chinese (36.7%), and Indian (16.7%). Most respondents (90%) attained a tertiary-level education and were employed. The mean (SD) duration of the illness is 21.3 (11.8) years.

**Table 1:** Characteristics of respondents (n = 30).

ID	Age (year)	Types	Duration of disease (year)	Impact on quality of life
A01	43	Scalp psoriasis	4	High impact
A02	45	Generalized pustular psoriasis	19	High impact
A03	46	Generalized pustular psoriasis	25	Mild impact
A04	51	Plaque psoriasis with PsA	36	Moderate impact
A05	32	Plaque psoriasis	19	Very high impact
A06	64	Plaque psoriasis	30	No impact
A07	46	Generalized pustular psoriasis	35	High impact
A08	29	Plaque and guttate psoriasis	17	High impact
A09	29	Plaque psoriasis	11	Very high impact
A10	27	Plaque and guttate psoriasis	5	High impact
A11	39	Plaque psoriasis	19	Moderate impact
A12	36	Plaque psoriasis with PsA	13	Very high impact
A13	63	Plaque and guttate psoriasis	50	High impact
A14	55	Plaque and guttate psoriasis	33	Moderate impact
A15	43	Plaque psoriasis with PsA	15	High impact
A16	47	Plaque psoriasis with PsA	15	Moderate impact
A17	44	Plaque psoriasis with PsA	30	High impact
A18	49	Plaque psoriasis with PsA	21	No impact
A19	32	Plaque psoriasis with PsA	26	Very high impact
A20	43	Plaque psoriasis	22	No impact
A21	43	Plaque psoriasis	35	Moderate impact
A22	29	Plaque and guttate psoriasis	11	Moderate impact
A23	67	Plaque and guttate psoriasis	10	No impact
A24	32	Plaque psoriasis	5	No impact
A25	60	Plaque psoriasis	39	Moderate impact
A26	43	Plaque and guttate psoriasis	32	High impact
A27	39	Plaque psoriasis	9	Mild impact
A28	48	Plaque and guttate psoriasis	15	High impact
A29	62	Plaque psoriasis	45	Mild impact
A30	36	Plaque psoriasis with PsA	19	Mild impact

Note: *ID* identification, *PsA* Psoriatic Arthritis.

Since the diagnosis was made, psoriasis has had a moderate to very high impact on QoL in 69% of respondents, which affects their daily activity (Table 2). Based on the DLQI, item 4 (the influence of skin on the clothes) had the most impact on QoL with a mean (SD) of 1.5 (1.10), followed by item 1 (itchy, sore, painful, or stinging sensation on the skin) with a mean (SD) of 1.4 (0.93) (Table 3).

**Table 2:** Impact on quality of life using Dermatology Life Quality Index (n = 30).

Disease Impact on Quality of Life	n	(%)
No impact	5	(16.7)
Mild impact	4	(13.3)
Moderate impact	7	(23.3)
High impact	10	(33.3)
Very high impact	4	(13.3)

**Table 3:** Description of Dermatology Life Quality Index items (n = 30).

	Item	Mean	(SD)
1	Over the last week, how itchy, sore, painful, or stinging has your skin been?	1.4	(0.93)
2	Over the last week, how embarrassed or self-conscious have you been because of your skin?	0.9	(1.03)
3	Over the last week, how much has your skin interfered with you going shopping or looking after your home or garden?	0.4	(0.49)
4	Over the last week, how much has your skin influenced the clothes you wear?	1.5	(1.10)
5	Over the last week, how much has your skin affected any social or leisure activities?	1.1	(1.02)
6	Over the last week, how much has your skin made it difficult for you to do any sport?	1.3	(1.12)
7	Over the last week, has your skin prevented you from working or studying? If “No”, over the last week, how much has your skin been a problem at work or studying?	0.7 0.6	(1.05) (0.85)
8	Over the last week, how much has your skin created problems with your partner or any of your close friends or relatives?	0.9	(1.02)
9	Over the last week, how much has your skin caused any sexual difficulties?	0.7	(1.11)
10	Over the last week, how much of a problem has the treatment for your skin been, for example, by making your home messy, or by taking up time?	1.2	(1.15)

### Impact of psoriasis on quality of life

The in-depth interview was summarised into six major themes: (i) physical devastation, (ii) emotional burden of the disease, (iii) disruption in social functioning, (iv) therapeutic hurdles, (v) cost-related hardship, (vi) behavioral adaptation (Table 4).

**Table 4:** Themes and subthemes of the lived experience of patients with psoriasis.

Themes	Subthemes
Physical devastation	Fashion compromise Chronic symptoms and pains Burden to others Mobility challenge Vicious cycle to trigger
Emotional burden of the disease	Emotional dysregulation Mental health struggles Unravelling self-worth Stress-induced flares Social anxiety
Disruption in social functioning	Relationship and intimacy challenge Future uncertainty Social stigma and rejection
Therapeutic hurdles and advancement	Gaps in the continuum of care The weight of lifelong treatment Intolerable side effects Advanced therapeutic agent
Cost-related hardship	Financial barriers to treatment Employment and income instability
Behavioral adaptation	Personal resilience and adaptation Advocacy and awareness Family, peer, and community support Financial support Holistic self-care approach

#### Theme 1: Physical devastation.

The first theme describes the physical devastation. It refers to the significant physical implications psoriasis has on respondents, including chronic pain, visible skin lesions, limited mobility, and reduced ability to perform daily activities. Findings related to physical devastation fell into five subthemes: Fashion compromise, chronic symptoms and pain, the burden to others, vicious cycle to trigger, and mobility challenges.

### Fashion compromise

Many respondents shared that they changed the way they dressed to hide visible skin lesions due to feelings of embarrassment and fear of being judged. These adjustments in clothing often lowered their self-confidence and led to social avoidance.

*“When it comes to clothing, I always choose dark-colored outfits. Any blood or pus will not be noticeable if I wear dark clothes. But if I wear something white, everything shows blood, pus, all of it. That is why I prefer black shirts. They help hide it”* (A10)

This choice was made to hide his disease, not only to avoid visible stains from blood or pus but also to reduce the emotional discomfort of being stared at or judged by others in public. One respondent tearfully recounted missing her best friend’s wedding because she could not find an outfit that covered her flare-ups.

*“Everything I tried on just made me feel disgusting, I did not want to be the one people stared at or whispered about.”* (A26)

### Vicious cycle to trigger

One respondent described how stress from her job consistently triggered psoriasis flare-ups, which in turn worsened her emotional state:

*“When I am stressed, the patches spread like wildfire. Then I start worrying about how bad I look, and that stress makes it even worse. It feels like I am trapped in a loop I cannot get out of.”* (A07)

This cycle left respondents feeling helpless and exhausted, highlighting how psoriasis affects both the body and emotions. Many respondents agreed that this cycle was hard to break. Another respondent shared how emotional distress from losing her beloved son led to one of the worst psoriasis flare-ups she had ever experienced:

*“When my son passed away, the grief was unbearable. That night, I went to bed heartbroken, and by morning, my skin broke out in painful pustules. They were all over me, like corn on the cob. The more I cried, the worse it got. I was in pain, inside and out. It felt like my sorrow was pouring through my skin.”* (A02)

### Burden to others

The constant skin flaking made respondents feel ashamed and burdensome to others. The flakes would fall onto beds, chairs, and floors throughout the day, requiring frequent cleaning that some could no longer manage alone. For those living with family members, this created a deep sense of guilt, as they felt their condition was causing extra work and discomfort for others. One respondent shared that he made the difficult decision to move out of the family home and live alone to avoid burdening his mother with the constant mess and symptoms (A05).

One respondent shared that she felt burdened because her skin kept shedding flakes onto the office carpet. The janitor had to vacuum more often, and she often heard complaints about the extra cleaning.. This made her uncomfortable at work and more aware of how her condition affected people around her (A14).

### Mobility challenge

Several participants shared how painful lesions on their feet, joints, or skin folds made simple movements difficult. Walking, climbing stairs, or standing for long periods became exhausting and painful. For those diagnosed with psoriatic arthritis, the symptoms became even more debilitating: joint stiffness, swelling, and pain severely limited their mobility. One respondent described feeling trapped in her body as flare-ups around the knees and ankles made every step a struggle (A04). This loss of physical freedom affected their independence and deepened feelings of frustration, helplessness, and emotional exhaustion. A few respondents described how their limited mobility led to frequent absences from work and made it difficult to carry out basic daily activities such as self-care, eating, and praying, eventually causing them to lose hope in managing their disease. One respondent described how limited mobility made even simple daily tasks challenging. He shared that dressing herself became difficult, often needing tools like a long stick to help remove her clothes. Bathing was also a struggle, as she could no longer reach her back or lift her arms properly to wash her hair, managing only to clean the sides of her head (A15).

### Chronic symptoms and pain

Respondent describes that itching, burning, cracked skin and pain affecting them day and night.. Many shared how the itching was so intense that it led to bleeding. Even routine activities like bathing, walking, or changing clothes became painful experiences due to skin sensitivity and the presence of open sores. Several respondents described it as a pain that never lets you forget you are sick.

*“When it is pustular psoriasis, the pain is unbearable. It feels like my skin is being scraped with a knife because the pus makes it so raw and painful.”* (A03)

*“It was a real struggle even just to go to the toilet. I went two weeks without proper sleep as a result. The itchiness was unbearable, and the pain made my skin feel burned. Every night, I ended up crying because I could not sleep.”* (A09)

#### Theme 2: Emotional burden of the disease.

The second theme describes the emotional burden of the disease and refers to the psychological distress that comes from living with a visible, chronic, and often misunderstood disease like psoriasis. This burden has been divided into five subthemes: emotional dysregulation, mental health struggle, unravelling self-worth, stress-induced flare and social anxiety. Over time, this emotional imbalance can worsen stress levels and even contribute to more flare-ups, creating a harmful cycle between the mind and the skin.

### Emotional dysregulation

The majority of respondents reported difficulty in controlling their emotions as a result of living with psoriasis. They described frequent mood swings, emotional outbursts, and moments of intense sadness, often triggered by new flare-ups or social interactions.

*“Sometimes I am fine, but when new symptoms appear where everyone can see, I suddenly get so angry or just break down crying. It is like I cannot control it.”* (A21)

The unpredictability of the disease and constant fear of judgment contributed to emotional instability and further impacted their psychological well-being. One of the respondents described how the emotion has taken a toll on her well-being.

*“It is really stressful. I get angry so easily because I cannot stand the heat. My skin just cannot take it. It makes me lose my temper over the smallest things. Sometimes, it feels like I just cannot accept what is happening to my body”.* (A10)

### Mental health struggles

Respondents in this study are juggling with their mental health while trying to cope with their condition. Several participants shared how the condition deeply impacted their mental well-being, manifesting as depression, hopelessness, and emotional exhaustion. One respondent described,

*“There were days I did not want to wake up. Not because of the pain, but because I was tired of fighting. Fighting the stares, the silence, the feeling that I am not normal. Psoriasis did not just take over my skin... it took over my mind.”* (A11)

Their feelings got worse during hospital stays. Another participant reflected,

*“During the darkest moments, thoughts of suicide often crossed our minds, especially when enduring persistent pain, lying in a hospital bed, and feeling completely alone.”* (A07)

This highlights how most respondents often felt emotionally overwhelmed and mentally exhausted, with psychological distress directly related to each episode of visible flare-ups.

### Unravelling self-worth

Many respondents often expressed deep emotional pain tied to feelings of rejection, unattractiveness, and loss of physical intimacy. Two respondents shared,

*“Sometimes I wonder if I am meant to spend my life alone. Even a massage feels like a reminder that I am ugly, unwanted, and no one wants to touch me.”* (A05)

*“It makes me feel sad because it reminds me that I am not someone people would want to be intimate with, that people do not want to touch me, do not want to have sex with me.”* (A10)

Psoriasis can affect self-worth and make intimacy harder. The fear of rejection, body shame, and perceived unworthiness not only contribute to psychological distress but also impact relationships and sexual health.

### Stress-induced flare

A large number of respondents described a clear and personal connection between their emotional stress and the worsening of their psoriasis symptoms. They expressed that stress was the primary cause of their flare-ups, while some observed that lesions became more widespread during stressful periods. This perceived link between stress and skin reactions created a constant cycle of worry, where emotional distress not only affected mental well-being but also directly intensified physical symptoms.

*“Because I work in engineering, the job is stressful and I do not get enough rest. That is when my psoriasis flares up badly”* (A03)

*“My major reason for flaring up is stress. Most of the time, when I am emotionally or mentally stressed, that is when I usually experience a flare-up.”* (A11)

### Social anxiety

This feeling refers to the fear of being judged, rejected, or negatively noticed by others in situations the respondents face.

*“Because of my skin, I tend to push people away before they even get the chance to know me. I assume they will judge me or want to avoid me. But sometimes, when they do get to know me, they do not even care about my skin. Still, my own insecurities make it hard to let people in.”* (A10)

*“Going to the swimming pool was really difficult for me. Wearing swimwear meant exposing my skin, and I could feel people staring or looking uncomfortable. That made me anxious and self-conscious, so I started avoiding those situations altogether.”* (A12)

Most respondents expressed feelings of anxiety when attending social events or visiting public places, fearing that their skin condition would influence how others perceived them.

#### Theme 3: Disruption in social functioning.

The third theme explained how the respondents’ skin condition affected their involvement in social activities. This disruption has given rise to three key subthemes: future uncertainty, relationship and intimacy challenges, and social stigma and rejection.

### Relationship and intimacy challenge

Most of the respondents have expressed how the disease has taken a toll on their relationships and intimacy with their spouse, family, and peers. Some respondents struggle with the lack of understanding and support from partners by expressing frustrations over their spouses’ inability to empathize with their pain or limitations, particularly during flare-ups. A respondent explained how her illness ultimately ended her marriage.

*“Because of my illness, I could not do things like cook or be intimate like before. My husband got frustrated and said I was useless. He did not understand my pain and just saw me as a burden. In the end, he left. The disease ruined our marriage”* (A11)

*“Because of my condition, my husband refuses to share a bed with me. He acts as if I am contagious. But nothing compares to the day my daughter asked me not to attend her school event. She was ashamed to be seen with me, her own mother. This has taken my family’s love and my place in my own home.”* (A07)

This showed how respondents experienced their skin condition impacting the relationship and intimacy due to their spouse’s rejection. Furthermore, persistent fear of looking sexually unattractive due to visible symptoms makes the relationship worse.

### Future uncertainty

Several respondents reported that their condition had affected their future aspirations, including ambition, career goals, and family planning. Some felt discouraged from pursuing success, believing their skin condition would limit their potential, while others feared the possibility of passing the disease on to their future children.

*“I do not see any future, I do not see any light at the end of the tunnel.”* (A17)

The respondent had given up on advancing in his career as the progression of his disease limited his ability to perform effectively.

*“I decided to stop at one child. Not because I did not want more but because I was afraid. What if she ends up like me? I do not want her to go through this pain. I am already saving up, just in case she ever needs treatment in the future.”* (A18)

### Social stigma and rejection

Almost all respondents shared experiences of rejection and stigmatization throughout their journey of living with the disease. Half of the respondents described experiencing rejection in public settings such as salons, spas, massage centres, and barber shops. One respondent recounted being asked to leave a massage centre due to his visible skin condition. Despite having visited the same place previously without issue, he was told they could no longer serve because the massage therapist felt uncomfortable. The experience left the respondent feeling rejected and deeply embarrassed, highlighting the ongoing stigma faced in public service settings (A10). One respondent shared an experience at a salon where the hairstylist refused to cut their hair, stating it was “too dirty” due to their skin condition (A12). They also mentioned losing some close friends over time, as not everyone could understand what they were going through. Some friends started to avoid them, which made them feel isolated.

#### Theme 4: Therapeutic hurdles and advancement.

The fourth theme describes the experiences and challenges of respondents during their treatment journey and the effects of the treatment. It also highlights how advancements in treatment have impacted them. It has arisen into four subthemes: gaps in continuum care, the weight of lifelong treatment, intolerable side effects, and advancement of therapeutic agents.

### Gaps in the continuum of care

Many respondents shared their frustrations about the difficulty in getting continuous care for their condition. Some mentioned that specialist clinics, such as dermatology, are only available on certain days, making it difficult to get regular appointments (A14). Others explained how they were transferred between different hospitals, which caused delays in their treatment. For example, one respondent had an appointment in October but was referred to another hospital and had to wait until January to be seen (A15). These changes also meant they had to repeat their medical history to new doctors each time, which was tiring and frustrating (A21). The long gaps between appointments were another concern, as some respondents felt their symptoms worsened before the next visit (A18). Overall, the system felt inefficient and made it harder for them to manage their condition properly.

### The weight of the lifelong treatment

Half of the respondents describe the burden of lifelong treatment of the disease. A few other respondents are concerned that the disease will not go away despite compliance with medication.

*“The last thing I want is for them to go through what I am dealing with. Psoriasis is not like a fever that goes away. It is a lifelong condition, and there is no clear end to the treatment. That uncertainty is what scares me the most”* (A19)

*“No matter what I do, my skin still does not improve sometimes. I control my diet and apply the cream regularly. I follow everything the doctor says, as well as any advice from others. But it still does not get better.”* (A21)

### Intolerable side effects

The most frequently reported experience among respondents was the challenge of tolerating the side effects of treatment. The majority of respondents had undergone multiple treatments to control their disease and improve their QoL. Ten respondents described serious physical effects, such as severe hair loss, cracked lips, dry and itchy skin, blurred vision and memory issues. One respondent was treated with methotrexate and steroids, which were commonly used but experienced long-term effects such as skin thinning, black marks, increased cholesterol levels and fears about liver and organ damage (A05). Another respondent even shared experiences about continuing medication during pregnancy, which led to miscarriage (A02). Despite trying both oral and topical treatments, many felt the relief was temporary and came at the cost of their physical comfort and emotional well-being. Five respondents shared that they have given up on the hospital treatment due to the side effects, thus changing to functional medicine.

### Advanced therapeutic agent

On the contrary, advances in medical technology have led to the development of new drugs known as biologics for treating psoriasis. These medications are given through subcutaneous injection, and fourteen respondents who received them described biologics as a “wonder drug.” They shared that the treatment helped remove the skin patches associated with psoriasis and regain their quality of life.

*“At that time, my skin was completely clear 100%, even more. It felt like I had my normal life back again”* (A05)

*“Yes, after starting biologics, it felt like I came back to life. It was like I got my life back.”* (A07)

Furthermore, this biologic agent has fewer side effects than other treatments prescribed to the respondents.

#### Theme 5: Cost-related hardship.

The fifth theme refers to the financial challenges experienced by respondents while managing psoriasis throughout their lives. This theme is further explained by two subthemes: financial barriers to treatment, employment, and income instability.

### Financial barriers to treatment

Most respondents describe their psoriasis treatment as having a negative financial impact. This is due to the cost required to obtain the best treatment for their lifelong skin condition. Some of the medication is very expensive, and a single tube of cream costs them several hundred ringgit. Biologic agents are one of the most expensive medications that need to be spent on. It is available, but not affordable to most respondents. One respondent shared that he had used the savings he had set aside for Hajj to pay for biological treatment, and as a result, had to give up his dream of going on the pilgrimage (A10). However, most respondents have received treatment from government hospitals; thus, most medication is subsidized except for biologic agents. Thus, those who can afford it will be able to get it. Many respondents acknowledge that psoriasis is a disease not covered by insurance; thus, the patients must bear all the treatment costs. One of the respondents recalled that she was denied the insurance due to psoriasis (A17). This adds to the number of barriers preventing the respondents from getting the best treatment.

### Employment and income instability

Psoriasis has affected the employment of several respondents, leading to income instability. They were not accepted in certain workplaces due to stigmatism, thus affecting their ability to have a proper income to support their medical expenses. One respondent said she needs to keep changing her job due to being unable to face the stigma at the workplace; thus, the salary is inconsistent to support her treatment (A16). Another respondent added that frequent visits for appointments and medical leave due to flares have resulted in the salary not being paid on the absent day (A07). A few respondents faced financial constraints as they had to spend more on treatment, leaving less for other necessities in their daily lives.

#### Theme 6: Behavioural adaptation.

The sixth theme refers to the respondent’s coping mechanism when dealing with the challenges throughout their psoriasis journey. This theme emphasizes five subthemes: personal resilience and adaptation, advocacy and awareness, family, peer and community support, financial support, and holistic self-care approach.

### Personal resilience and adaptation

In this study, most respondents slowly learned to accept their condition and find ways to live with it. Despite the challenges, many developed a stronger mindset, changed their attitudes, and found positive ways to cope with their daily struggles.

*“I told myself, I do not want psoriasis to control me. I will not let it limit what I can do. I just have to stay strong and be resilient. You need to build that inner strength to keep going.”* (A06)

*“When I think about when it all started and where I am now, it does not really bother me anymore. I know I am still healthy, even with psoriasis. I am still alive, breathing, and having time with my son and family. I can still go out, travel, and enjoy life. I am still here, still doing things.”* (A11)

Many respondents believed that maintaining a positive mindset helped them manage their psoriasis and gave them more confidence when involved with society.

### Family, peer and community support

Strong network support plays an important role for individuals living with psoriasis. With good support, the journey with psoriasis is bearable and helps them adapt earlier and feel more appreciated.

*“On the other hand, this experience has strengthened my relationship with my family. It really opened my eyes to how much they have supported me throughout my journey with psoriasis and psoriatic arthritis”* (A19)

*“They accompanied me to medical appointments and supported me throughout the treatment process. They were fully present with me every step of the way. I also informed my employer at the time, and he was very understanding and supportive.”* (A24)

### Financial support

In this study, we found that the major subthemes that help individuals cope are obtaining financial support for better treatment access. Most respondents expressed a strong desire to have access to the best available treatment in hopes of improving their condition. While the government provides subsidized treatment for psoriasis, biologic therapy is considered one of the most effective options that is not covered. As a result, only a few respondents could access it through hospital social welfare programs or limited sponsorships from pharmaceutical companies. The remaining patients had to bear the cost, making the treatment financially burdensome. Few respondents were able to receive financial assistance through medical zakat. With a referral letter provided by their doctor, they applied for support and successfully obtained aid (A25). One respondent shared that the Public Service Department covered his treatment as a government servant entirely. This support allowed him to access necessary medications without financial burden, which he felt was a great relief compared to others who had to pay out of pocket (A30).

### Advocacy and awareness

Increased awareness through advocacy has greatly helped many respondents cope with the disease. One respondent shared how she actively sought out others with similar experiences and formed a supportive community. She started a WhatsApp group and later formed an association to raise awareness. She encouraged others not to feel ashamed and helped educate the public that psoriasis is not contagious and needs proper treatment (A17). Another respondent described her journey toward self-acceptance by participating in beauty pageants and proudly displaying her psoriasis scars on public platforms. She highlighted the value of online support groups where knowledge and encouragement are continuously shared, helping patients feel connected and empowered (A17).

### Holistic self-care approach

A holistic self-care approach means caring for the body, mind, and spirit. It includes spiritual practices like prayer and meditation to stay calm and positive, along with healthy lifestyle changes such as eating well, exercising, managing stress, and avoiding triggers like hot weather and smoking.

*“I started paying more attention to my diet and lifestyle. I cut down on certain foods, try to stay active, and manage my stress better. It is not easy, but I can see it helps reduce the flare-ups and makes me feel more in control.”* (A28)

*“I have learned to accept this as part of my journey. I believe there is a reason for everything, and this is a test from God. So, I have faith and continue to pray. It gives me peace and strength to go through it”* (A01)

## Discussion

This current qualitative study aimed to explore the lived experience of patients with psoriasis in Malaysia, specifically their QoL. This study revealed that psoriasis has a significant impact on QoL, aligning with several studies related to QoL in patients living with psoriasis [6,13,20,21].

In interpreting the findings of this study, the biopsychosocial model is a guiding framework to explain how the biological symptoms of psoriasis intersect with patients’ emotional responses and social challenges [22]. Moreover, this model was developed to emphasize interconnections between the three components.

From a biological perspective, most respondents commonly reported physical burden characterized by persistent itching, scaling, pain, and visible skin lesions. It is correlated with item 4 (itchy, sore, painful, or stinging sensation on the skin) in DLQI with the most impact on QoL, which highlights the physical devastation theme, particularly on chronic symptoms and pain subthemes. Similar findings were reported in a study of over 20 patients from a general specialist dermatology practice in Taiwan that described the chronic symptoms [23]. These symptoms often disrupt daily functioning and demand lifestyle modifications, such as selecting clothing to conceal affected areas and avoiding certain physical activities [24]. Apart from individual discomfort, psoriasis can indirectly affect others in the surroundings, particularly due to the physical presence of skin flakes and scales. Respondents in this study often experience a burden on others, knowing that excessive shedding may cause discomfort or inconvenience to those around them, especially in shared spaces such as the home or workplace. This aligns with previous qualitative findings that shedding skin flakes has caused people discomfort [21].

Additionally, a few respondents had joint stiffness, which caused pain that contributed to reduced mobility [20]. This aligns with the finding in the DLQI, which shows a high mean score on items related to leisure and sports activities. Furthermore, psoriasis is a systemic inflammatory condition that can result in significant physical morbidity and function [25]. Effective symptom control is crucial for achieving skin clearance and maintaining patients’ physical QoL. If left unmanaged, these biological symptoms may contribute to significant psychological distress [26].

While exploring the psychological dimension, psoriasis imposed significant emotional turbulence across nearly all respondents in this study. They reported feelings of embarrassment, low self-esteem, and emotional distress, which can lead to depression, often exacerbated by the chronic nature of the disease and its visibility [6]. This is supported by item 2 (embarrassed or self-conscious) in the DLQI, which aligns with the emotional burden theme. A systematic review reported that the prevalence of depression among psoriasis patients ranges widely, with higher rates observed in those with more severe disease [27]. In this study, respondents were struggling with mental health and their self-worth. Stress is one of the significant issues that has been reported to induce flares and cause social anxiety. These experiences reflect a well-documented phenomenon in psoriasis, where stress activates inflammatory pathways that contribute to disease exacerbation. Psychological stress can influence immune function and skin barrier responses, significantly influencing both the onset and worsening of psoriasis [28]. These sentiments align with the findings in the DLQI on the item related to feelings, which show an alarming mean score representing their psychological burden.

Interpreting the social implications of psoriasis reveals how stigma and relationship strain contribute to the overall disease burden [29]. Psoriasis is frequently misunderstood, with misconceptions linking it to poor hygiene and contagiousness [6]. In this study, respondents described their experiences of social stigma, rejection, and challenges in intimate relationships. It is supported by item 8 (problems with partner, close friends, or relatives) on the DLQI, which yields a significant score, and raises the theme of disruption in social functioning. Psoriasis has negatively affected participants’ sexual well-being, largely due to feelings of unattractiveness and sexual undesirability. This finding mirrors a study conducted among 51 psoriasis patients at Croatia’s Dermatology Department of the University Hospital [6]. Additionally, they expressed deep concerns about the long-term impact of their condition, particularly the possibility of passing it on to their children [21]. Furthermore, these social impacts of psoriasis include reduced productivity and financial hardship [6].

The dual nature of psoriasis treatment is marked by therapeutic challenges and the promise of newer, more effective options. While some respondents report improvement with modern biologics, others struggle with long-term adherence, uncertainty about outcomes, and undesirable side effects from conventional treatments. The considerable time required for personal care and medication application had a notable emotional impact on patients, showing a strong correlation with feelings of anger and frustration. This is illustrated in item 10 (treatment problems to skin) in the DLQI and correlates with the theme of therapeutic hurdles and advancement. Several patients’ satisfaction with available therapies was moderate; those receiving biologic treatments reported the highest satisfaction levels compared to individuals using topical agents, phototherapy, or conventional oral therapies [30]. This finding is consistent with a qualitative study involving 20 respondents, which reported similar effects after undergoing biologic therapy [23].

Financial strain due to treatment costs and employment instability further compounds these issues. The economic burden of psoriasis often includes expenses related to insurance, hospitalization, medical treatments, inpatient and outpatient care, prescription and over-the-counter medications, as well as indirect costs such as reduced productivity and work absenteeism [31]. Few respondents were denied insurance coverage, while others faced limited reimbursement or high out-of-pocket expenses despite having insurance, as psoriasis was not listed among the covered conditions, resulting in a financial burden. Directly, respondents were affected by hospital admission expenses, outpatient charges, and transportation charges [32]. In addition, despite the availability of effective treatments, respondents expressed concerns about the long-term financial sustainability of managing their condition, as current therapies did not always provide satisfactory long-term solutions [6].

Further understanding of behavioural adaptation after going through the impact of psoriasis on QoL, many respondents described modifying their routines and habits to manage flare-ups and maintain QoL [23]. Examples included using fragrance-free products, adjusting their diets, wearing specific clothing, and maintaining strict skincare routines. Many patients found ways to motivate themselves during their challenging treatment journey and saw managing psoriasis as part of everyday life. Over time, they learned to face the condition, accept it, deal with the challenges, and eventually make peace [23]. A few respondents in this study reported that avoiding hot, humid environments and incorporating daily meditation helps reduce flare triggers. These strategies reflect a proactive, adaptive approach to living with psoriasis [10].

Respondents in this study also emphasize that strong support from family, peers, and the community has a significant impact on their ability to cope. This finding aligns with the qualitative meta-synthesis of 21 studies involving patients with psoriasis, highlighting the important role of family and community support in mitigating the psychosocial burden of the disease. In their study, patients shared that emotional support from family members and help with treatment routines helped ease feelings of shame and isolation. Through peer groups and public awareness efforts, community support also played a crucial role in reducing stigma and fostering a sense of belonging among individuals living with psoriasis [33]. Furthermore, this aligns with behavioral coping strategies: adaptive behaviors, including self-monitoring and lifestyle adjustments, play a key role in maintaining well-being [34].

Most themes were consistent with the DLQI findings. However, although respondents described how psoriasis affected their daily activities due to pain and skin changes, this was not reflected in item 3 (daily activity) of the DLQI, which reported the lowest mean score. This led to the emergence of a new theme on behavioral adaptation, particularly on subthemes of personal resilience and adaptation.

This study is not without limitations. As all participants were recruited from members of the Psoriasis Association of Malaysia, the sample may not fully represent the broader psoriasis population in Malaysia. This selective recruitment may introduce bias because the members might have different experiences, access to resources, or support systems than those not affiliated with such groups. Furthermore, participants were not differentiated by clinical severity, disease duration, comorbidities, or demographic subgroups, and their relation to the QoL. These variables may influence their perceptions of illness and coping strategies. However, the qualitative approach offered rich and unique insights into the experiences of patients living with psoriasis in Malaysia.

Future studies involving a larger and more diverse sample that integrates the relation between clinical severity and demographic subgroups from multiple settings are recommended to provide broader insights into the experiences of individuals living with psoriasis. Research on long-term psychosocial outcomes can also help in understanding and managing the disease and the patient’s QoL. Additionally, it is highly recommended to address the importance of mental health care in conjunction with physical symptoms. If left neglected, this will further lead to social dysfunction. Besides, public education is crucial to raise awareness that psoriasis is not a contagious disease, which could help reduce the stigmatization of patients.

## Conclusion

Based on experience explored, living with psoriasis involves ongoing challenges such as physical discomfort, psychological distress, social stigma, and, not to forget, the challenges in treatment and financial burden. Using the biopsychosocial model as a framework, this study emphasizes the need for a holistic approach to psoriasis care that addresses medical treatment alongside emotional support and social empowerment to improve patients’ QoL. Behavioral adaptation is helpful in coping with the condition; however, it will be more effective with the support of the surrounding community in raising awareness about the disease burden. Thus, understanding the impact of the disease holistically is the key to improving the outcomes.
